# Supplementary figures and images for: Mapping the dynamics of visual feature coding: Insights into perception and integration
Source: PLoS Comput Biol. 2024 Jan 8;20(1):e1011760. doi: 10.1371/journal.pcbi.1011760 (PMC10798643; doi:10.1371/journal.pcbi.1011760)

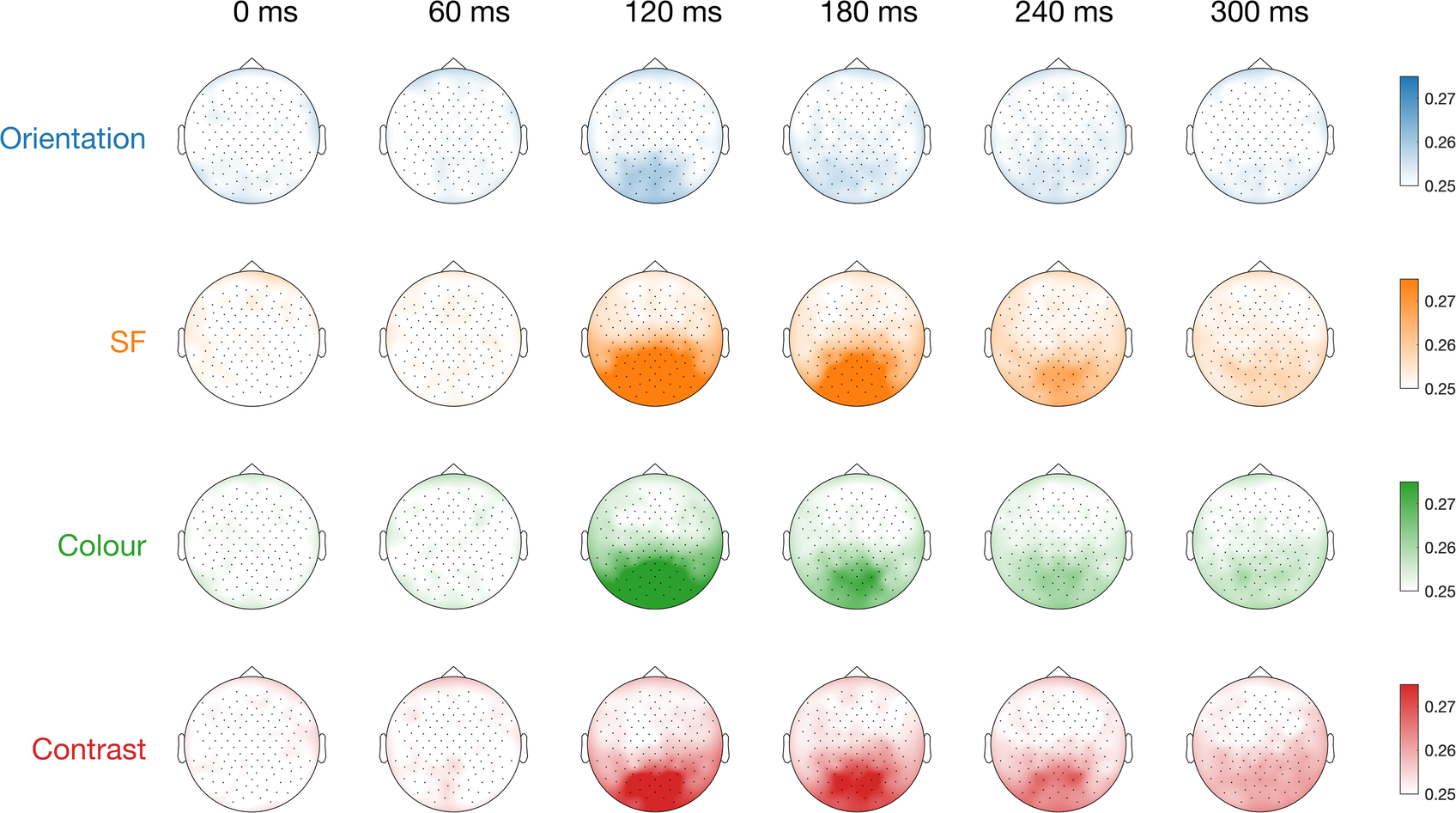

Supplement: S1 Fig — (TIFF) [file pcbi.1011760.s001.tiff]

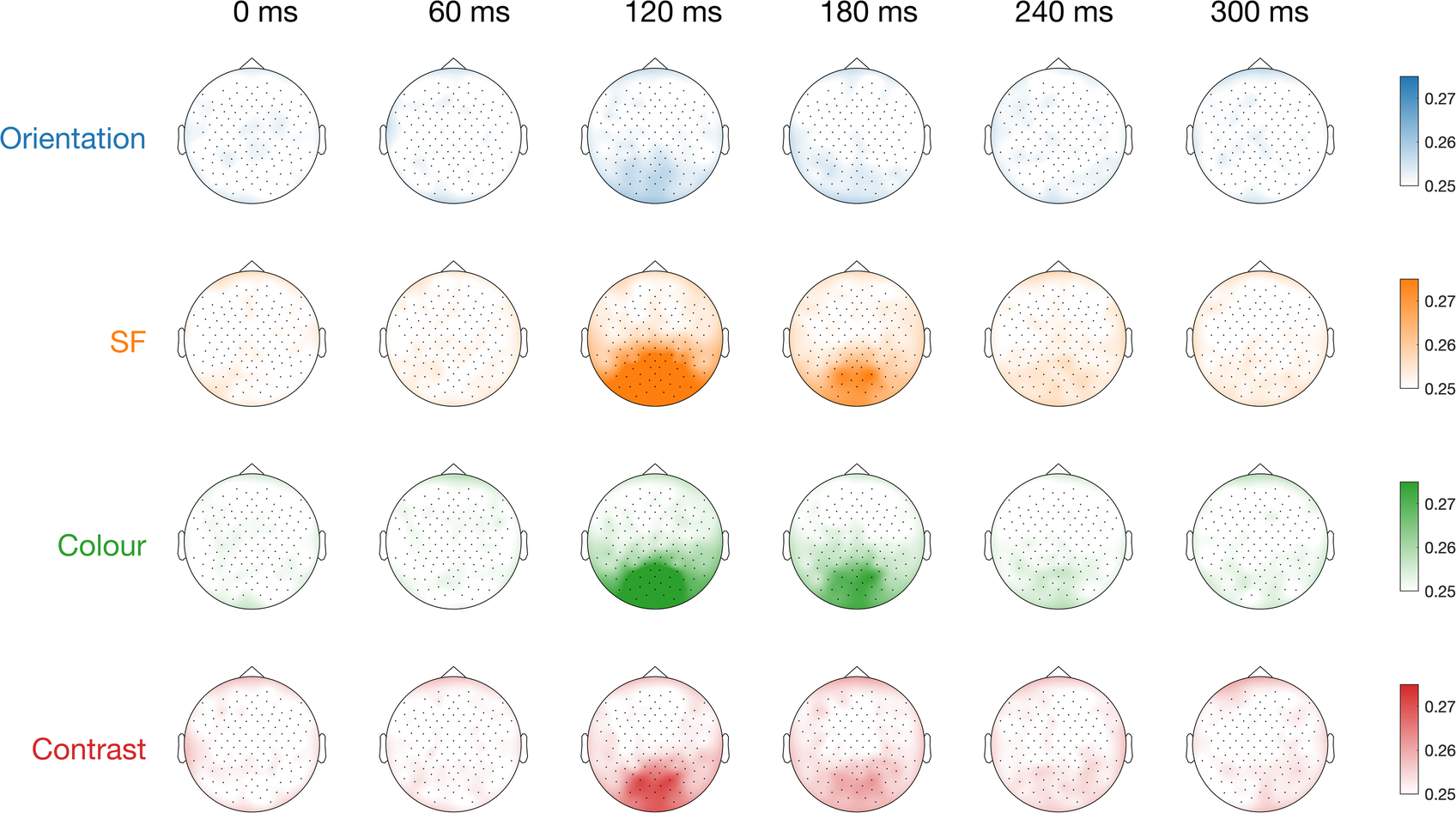

Supplement: S2 Fig — (TIFF) [file pcbi.1011760.s002.tiff]

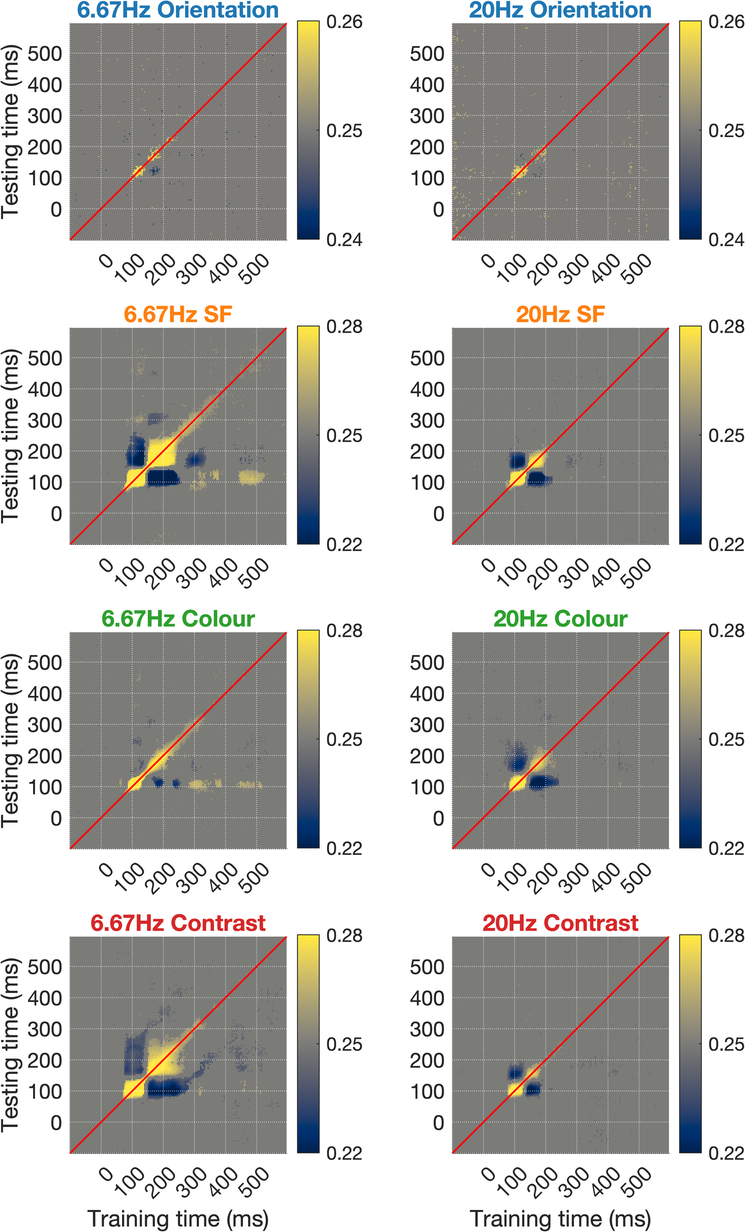

Supplement: S3 Fig — (TIFF) [file pcbi.1011760.s003.tiff]

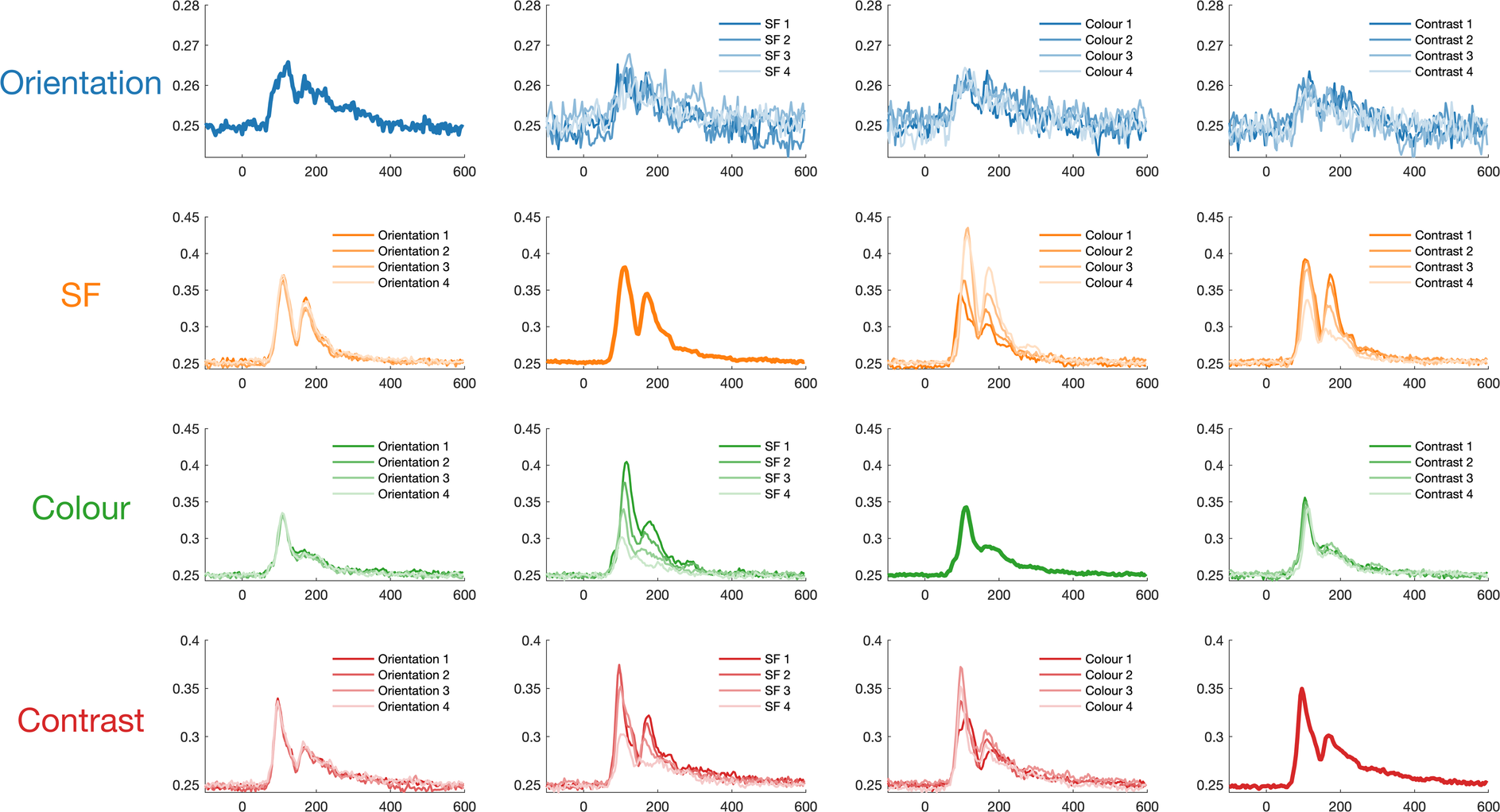

Supplement: S4 Fig — Plots on the diagonal show overall decoding for that feature when including all trials. (TIFF) [file pcbi.1011760.s004.tiff]

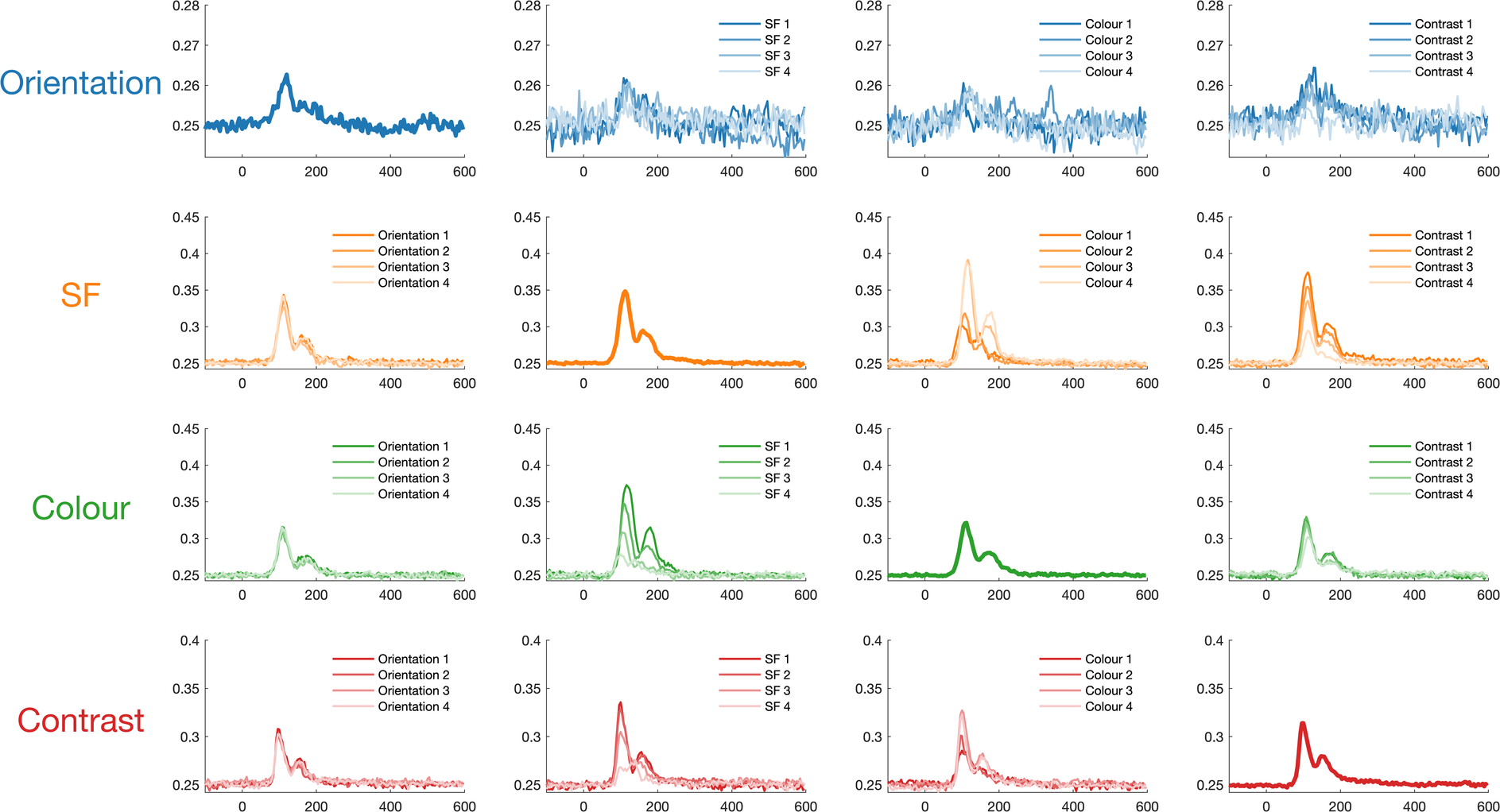

Supplement: S5 Fig — Plots on the diagonal show overall decoding for that feature when including all trials. (TIFF) [file pcbi.1011760.s005.tiff]

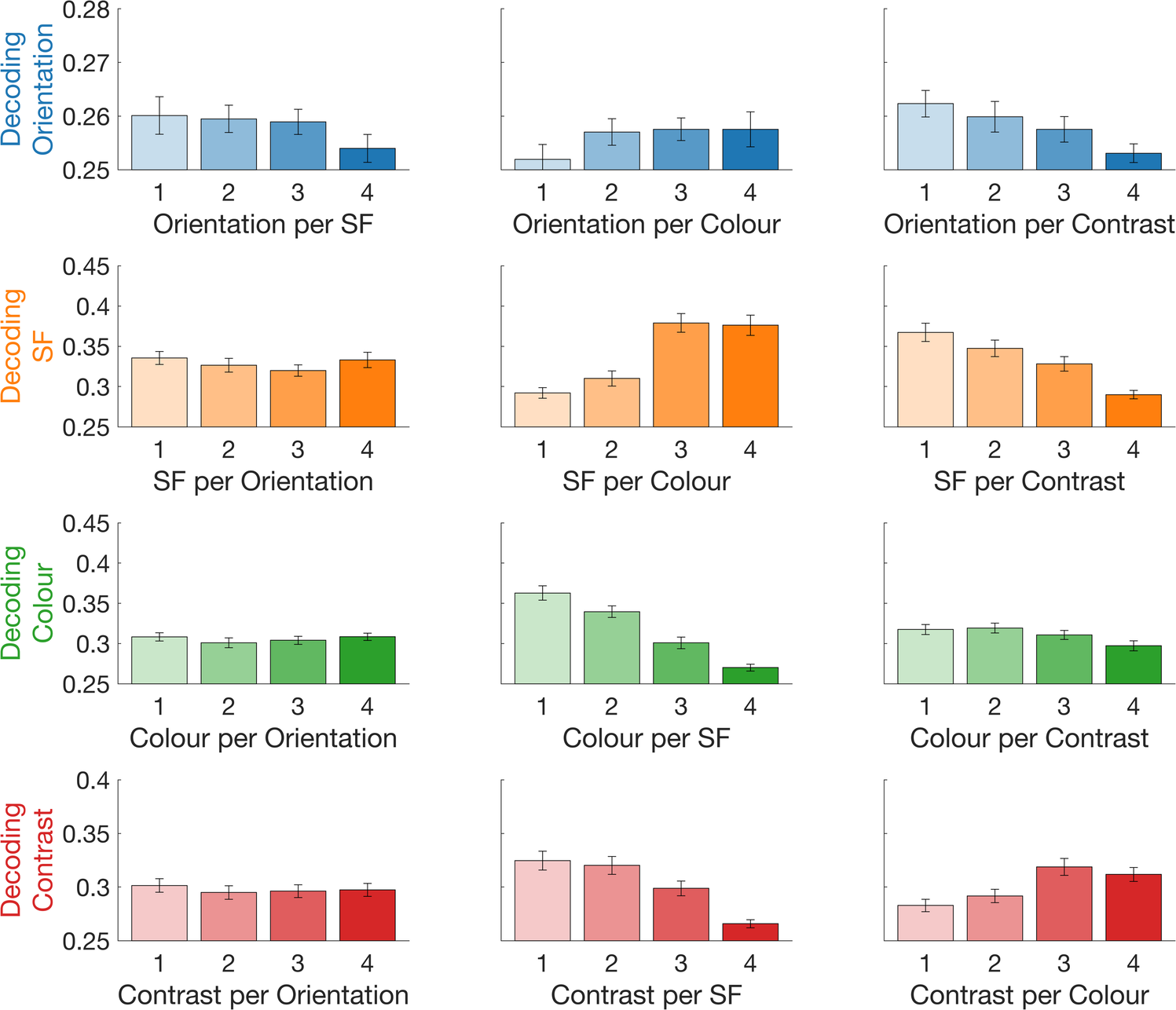

Supplement: S6 Fig — (TIFF) [file pcbi.1011760.s006.tiff]

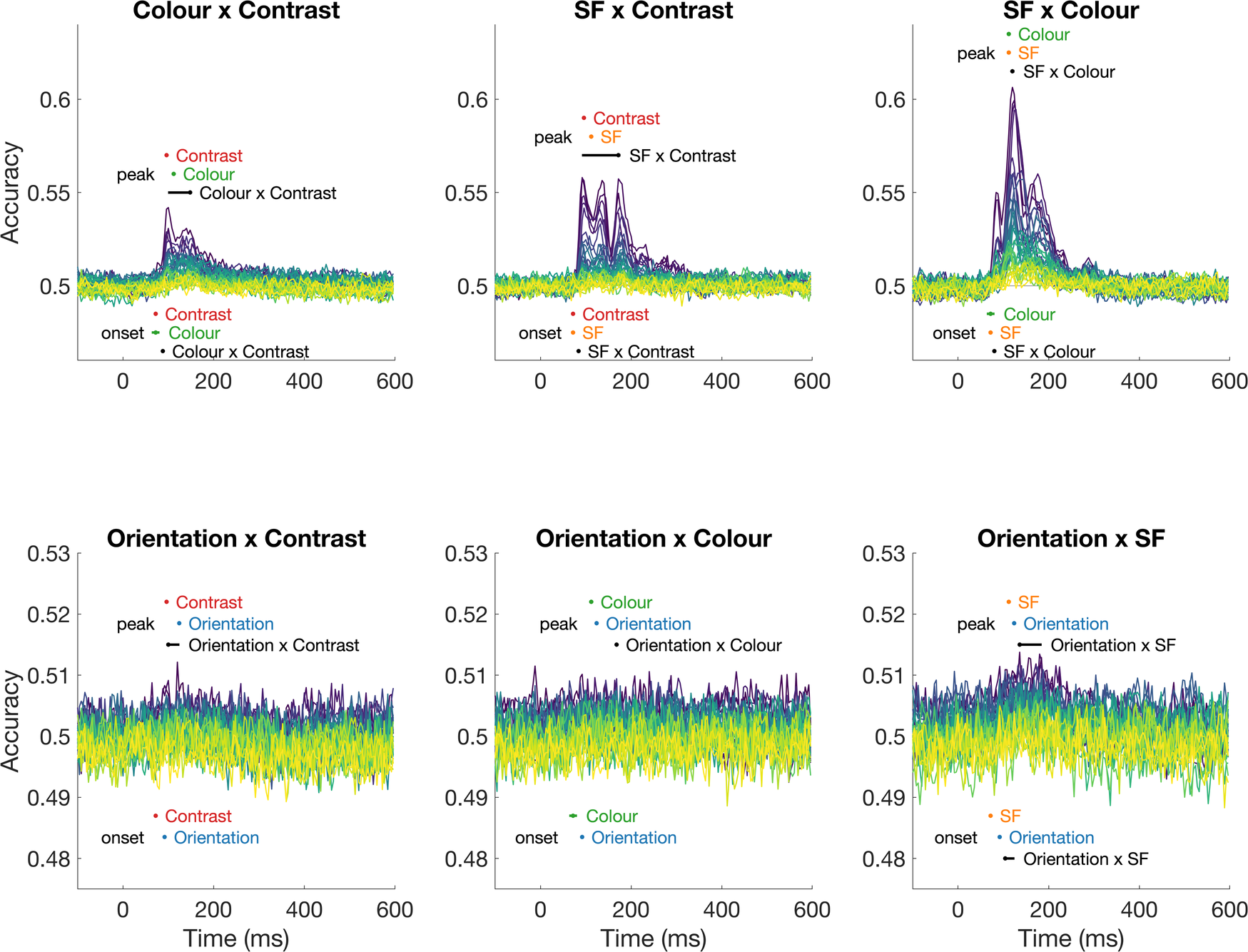

Supplement: S7 Fig — Within each plot, it is evident that the different contrasts varied in decoding performance, but were consistent in terms of the dynamics (e.g., peaks). Onsets and peaks of individual features and mean conjunctions are plotted with 95% confidence intervals; onsets are below the chance level and peaks are above. Note the different y-axis scales per row. (TIFF) [file pcbi.1011760.s007.tiff]

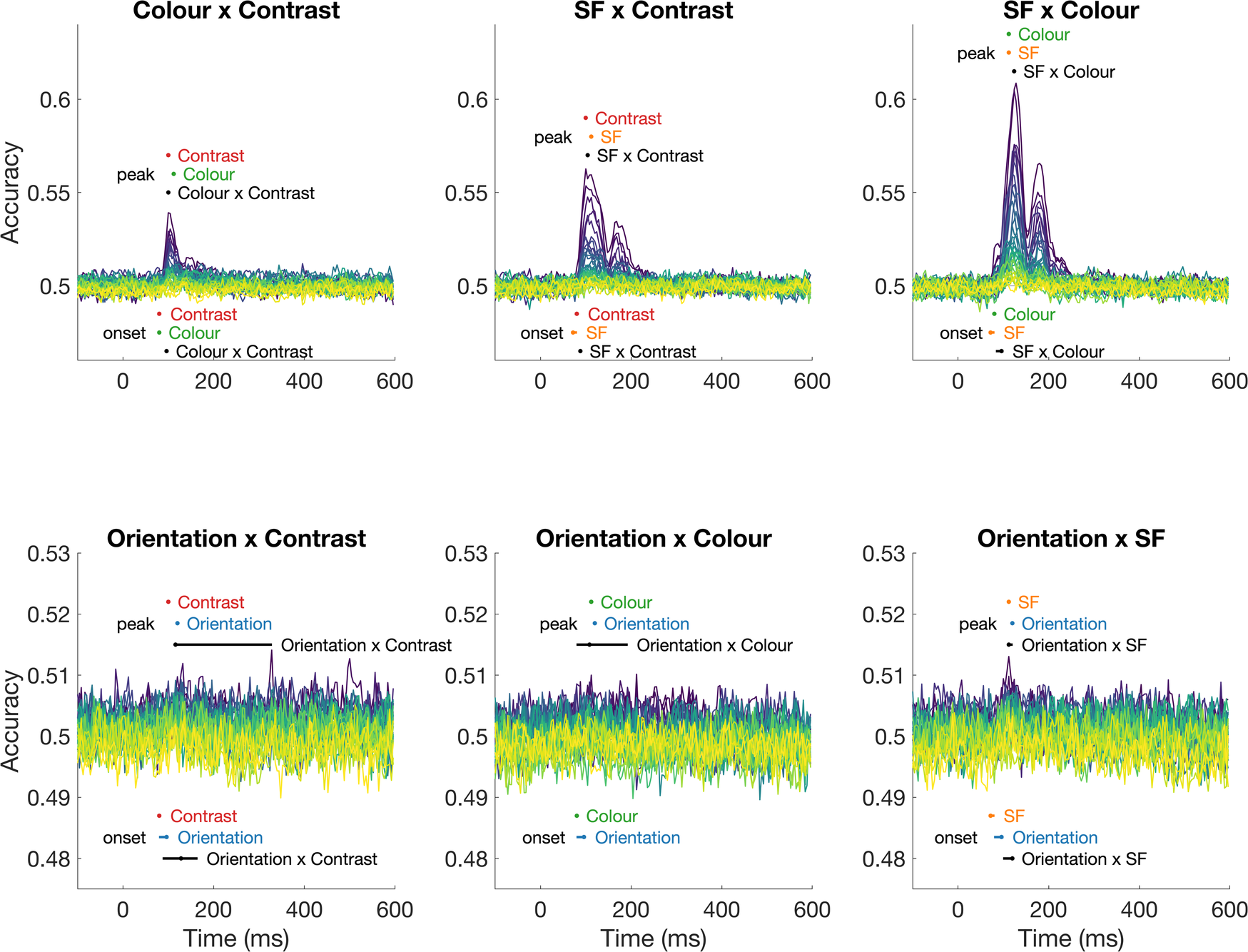

Supplement: S8 Fig — Within each plot, it is evident that the different contrasts varied in decoding performance, but were consistent in terms of the dynamics (e.g., peaks). Onsets and peaks of individual features and mean conjunctions are plotted with 95% confidence intervals; onsets are below the chance level and peaks are above. Note the different y-axis scales per row. (TIFF) [file pcbi.1011760.s008.tiff]
