# Supplementary material for: Mapping the dynamics of visual feature coding: Insights into perception and integration
Source: PLoS Comput Biol. 2024 Jan 8;20(1):e1011760. doi: 10.1371/journal.pcbi.1011760 (PMC10798643; doi:10.1371/journal.pcbi.1011760)
Supplement: Attachment — Submitted filename: 2023-07-10_peerReview.docx [file pcbi.1011760.s009.docx]

In this study the authors show the time-resolved decoding of four important visual features and their interactions. They show that each feature (contrast, orientation, spatial frequency and color) can be decoded from the EEG after ~90ms with some notable differences between features. They further perform interesting analyses regarding interactions and conjunctions, time-generalization and even perceptual similarity. They inspired interesting new questions for me, both on a methodological, and brain-conceptual level. I think their study is well executed, well powered and well written and I thoroughly enjoyed reading it.

The authors provide both data and code, thanks very much for this. The data was incredibly easy to use! I did not perform a direct replication, but used a partial implementation of back2back regression, to check their results for robustness. I could replicate the double peak (for color and contrast, surprisingly not for SF), the lack of orientation decoding and replicated the interactions (relating to the compound analysis here – but as encoding-model style multiplicative interactions). Further details in the analysis are outside the scope of the current peer-review (I ran out of time 😉), but if the author is interested I am happy to share. I did not perform a full replication of the study, thus I cannot claim fully checking for reproducibility.

I have some further small remarks that the authors most likely can address without a major revision.

**Strengths**

- **High power, well designed study**
- **Clear plots, open data, open code**

**Weaknesses**

- No weakness I found particularly noteworthy

Below you can find all of my comments.

Signed, Benedikt Ehinger

**Limitations of my review**

- **I feel pretty confident on most topics throughout my review**
- **I don’t have much experience with RDMs**

**Major**

**Problem Conjuntion analysis**: The paper states: “ Because the classes could not be distinguished based on any one feature alone, above chance classification for this analysis reflects unique neural responses to the *combinations* of different pairs of features, **indicating the presence of feature binding**.

” (L.168 – highlight myself).
I am not fully convinced of that last bit. Imagine a very contrived scenario, where each feature x feature level combination projects only to a dedicated electrode – then everything can be easily decoded by the pairwise classifiers. But by construction, there is no feature binding at all. Of course this is just a thought experiment, and the actual activity will never be that nicely distinguishable – but I lack intuition in what ways such an analysis could really distinguish between feature binding (that is, a new code for a specific combination) and the overlap of level-specific activity.
Taken together, for me the overall embedding of such conjunction analyses in a conceptual framework is missing. In the Paper Taylor & Xu that the paperrefers to in this context, it is slightly different. They have the additional assumption that due to their measuring of interleaved conjunction blocks in fMRI, this smears over the patterns over time-points (due to BOLD), enforcing that you cannot decode a single feature alone (I hope I got it right, not 100% sure). If I understand correctly, with EEG we would not be in the same situation.
But even in Taylor & Xu, if you have two voxels that are selectively active only for green or for red, and two for orientation 1 or 2, (that is, 4 voxels in total), you should be able to decode any pairwise feature combination without needing any underlying interaction.


**Minor**

- L23 “between neural coding and behaviour was evident from initial processing stages, signifying that the fundamental features, not just their interactions, are crucial for perceptual experience.“ – I am not convinced that this conclusion is following from this data. They could just be a remnant, without being necessary.
- Topgraphical importance maps – some more details are necessary here, what was the size of the searchlight kernel, how was feature importance defined (accuracy?). In Fig. 2A/B. A scale is missing, (if accuracy, that is easy then, if A.U. –maybe still interesting comparing the four different classifiers/features). Maybe it is necessary to mention that these are not inversions of the classifier – I got confused and at first assumed differently (Looking back, I didn’t read the caption properly, maybe more subheadings instead of only A/B could be helpful to map it better?)
- Figure 1B – the generalization maps are quite small - I would also push them to the same colorscale, requiring only one scale + freeing up space to enlarge them (and making them more comparable). It is unclear to me whether they were additionally masked by significance
- C1/P100 – C1 and P100 are quite different, I found it surprising to be seen in the same sentence. I don’t think the authors can make strong claims about C1, their timing is omparably late, and C1 (origin likely in V1) is typically identified via cruciform model / peripheral location of stimulus
- L294: I did not understand the logic of this argument. The idea is to explain why in MEG we don’t see a second bump. But being able to measure frontal-alpha (or not, as apparently in MEG), should not matter in this case, given that the weights in Fig 1B are all strongly occipital. Maybe the authors could clarify this passage.
- In L351 the paper argues that the orientation effect is so small, maybe because orientation is not an important feature. What I was missing was, that it could just be quite hard to measure in EEG due to cancellations within the fusiform gyrus. Thus, if you take the cruciform model, maybe activity over hemispheres (and top/bottom) cancels each other out, making it unmeasureable. It could then be, that showing a rotated stimulus only within one quadrant, that we can decode orientation quite fine – simply not if it is extended over the whole screen. Not sure what the authors can do with this, maybe there is some prior research on it?
- In relation to odd-one-out-triplet, I think this paper might be of interest <https://jov.arvojournals.org/article.aspx?articleid=2783900> (no expectation to include/cite that paper!)
- L451: Luminance levels (and environment) level might be interesting to some researchers
- L478: What monitor was used?
- In general, I could not see in the code or paper that the monitor delay was adjusted. This is quite important given the discussion of all the onset-timing-effects. Same is true for potential filtering delays.
- L488: Targets occurred more often in one SOA condition to the other, is that correct? Maybe making this transparent, if true, is helpful to some.
- SOA was confusing to me, as asynchronisity implied to me a jitter. Stimulus-Onset-Interval might be clearer?
- L494: Quite some details on the used filter is missing.
- There is no correction/control for eye-movements, even though in some experiments stimulus conditions can be decoded from fixational eye movements alone. I don’t particularly think this is a big issue here, given the feature importance showing little weight in frontal
  channels – but something worth discussing. E.g. Mostert showed this in MEG with orientation effects <https://www.ncbi.nlm.nih.gov/pmc/articles/PMC6179574/> .
- The circular nature of orientation was not used in this study, potentially leading to lower than possible decoding abilities.

One Question that was raised during reading (outside of scope of review – but still interesting to me, please ignore if you don’t have time to engage!)

- The n+1 / n+2 decoding ability, are these sequence effects? That is, if sf = 1 is followed by sf = 1, can we decode the latter one better or worse? I could envision it going both ways. In general, further I wonder if the “temporal code” changes (similar to evoked/sustained response of the cortex – see e.g. <https://doi.org/10.1016/j.celrep.2023.112614> )
