# Supplementary material for: Mapping the dynamics of visual feature coding: Insights into perception and integration
Source: PLoS Comput Biol. 2024 Jan 8;20(1):e1011760. doi: 10.1371/journal.pcbi.1011760 (PMC10798643; doi:10.1371/journal.pcbi.1011760)
Supplement: Attachment — Submitted filename: features_response_to_reviewers.docx [file pcbi.1011760.s010.docx]

We thank the Reviewers for their well-considered feedback. We have made substantial changes to the manuscript to address the comments and believe the manuscript is considerably stronger as a result. Our responses to each of the comments are listed below and changes in the manuscript are highlighted in yellow.

**Reviewer 1**

In this study the authors show the time-resolved decoding of four important visual features and their interactions. They show that each feature (contrast, orientation, spatial frequency and color) can be decoded from the EEG after ~90ms with some notable differences between features. They further perform interesting analyses regarding interactions and conjunctions, time-generalization and even perceptual similarity. They inspired interesting new questions for me, both on a methodological, and brain-conceptual level. I think their study is well executed, well powered and well written and I thoroughly enjoyed reading it.

The authors provide both data and code, thanks very much for this. The data was incredibly easy to use! I did not perform a direct replication, but used a partial implementation of back2back regression, to check their results for robustness. I could replicate the double peak (for color and contrast, surprisingly not for SF), the lack of orientation decoding and replicated the interactions (relating to the compound analysis here – but as encoding-model style multiplicative interactions). Further details in the analysis are outside the scope of the current peer-review (I ran out of time 😉), but if the author is interested I am happy to share. I did not perform a full replication of the study, thus I cannot claim fully checking for reproducibility.

Thank you, we are happy to hear you found it easy to use the data. We found a recent preprint that used the current open data for a new analysis too, and we are glad that the dataset seems useful for studying new research questions (https://doi.org/10.1101/2023.07.09.548296).

I have some further small remarks that the authors most likely can address without a major revision.

**Strengths**

·	**High power, well designed study**

·	**Clear plots, open data, open code**

**Weaknesses**

·	No weakness I found particularly noteworthy

Below you can find all of my comments.

Signed, Benedikt Ehinger

**Limitations of my review**

·	**I feel pretty confident on most topics throughout my review**

·	**I don’t have much experience with RDMs**

**Major**

**1.1 Problem Conjunction analysis**: The paper states: “ Because the classes could not be distinguished based on any one feature alone, above chance classification for this analysis reflects unique neural responses to the *combinations* of different pairs of features, **indicating the presence of feature binding**.” (L.168 – highlight myself).
I am not fully convinced of that last bit. Imagine a very contrived scenario, where each feature x feature level combination projects only to a dedicated electrode – then everything can be easily decoded by the pairwise classifiers. But by construction, there is no feature binding at all. Of course this is just a thought experiment, and the actual activity will never be that nicely distinguishable – but I lack intuition in what ways such an analysis could really distinguish between feature binding (that is, a new code for a specific combination) and the overlap of level-specific activity.
Taken together, for me the overall embedding of such conjunction analyses in a conceptual framework is missing. In the Paper Taylor & Xu that the paper refers to in this context, it is slightly different. They have the additional assumption that due to their measuring of interleaved conjunction blocks in fMRI, this smears over the patterns over time-points (due to BOLD), enforcing that you cannot decode a single feature alone (I hope I got it right, not 100% sure). If I understand correctly, with EEG we would not be in the same situation.
But even in Taylor & Xu, if you have two voxels that are selectively active only for green or for red, and two for orientation 1 or 2, (that is, 4 voxels in total), you should be able to decode any pairwise feature combination without needing any underlying interaction.

We appreciate that the conjunction analysis was not as clear as it should be. In our conjunction analysis, if feature combinations are just an additive response then we should not be able to decode between groups of stimuli containing the same features but in different combinations. Our ability to decode specific stimulus combinations indicates that feature conjunctions cause nonlinear responses. From our perspective, if every feature x feature level combination projects to a different electrode, as in your example, that would indicate the presence of feature binding. Without binding, the combinations should just be a sum of the individual features themselves and not project to different electrodes.

To explain another way, in our analysis, the classifier is using a linear boundary between classes so it cannot solve the XOR problem. Therefore we conclude that the brain must be solving the XOR for us to be able to decode it. We note also that Reviewer 3 thinks it is a good analysis (see point 3.3). We have tightened up the explanation of the methods and conclusions in the manuscript to make the conjunction analysis clearer.

**Minor**

**1.2** L23 “between neural coding and behaviour was evident from initial processing stages, signifying that the fundamental features, not just their interactions, are crucial for perceptual experience.“ – I am not convinced that this conclusion is following from this data. They could just be a remnant, without being necessary.

Thank you, we agree this conclusion should be tempered. We have changed to “signifying that the fundamental features, not just their interactions, contribute to perceptual experience”.

**1.3** Topgraphical importance maps – some more details are necessary here, what was the size of the searchlight kernel, how was feature importance defined (accuracy?). In Fig. 2A/B. A scale is missing, (if accuracy, that is easy then, if A.U. –maybe still interesting comparing the four different classifiers/features). Maybe it is necessary to mention that these are not inversions of the classifier – I got confused and at first assumed differently (Looking back, I didn’t read the caption properly, maybe more subheadings instead of only A/B could be helpful to map it better?)

We have added details to the method regarding the searchlight decoding, and included more detail in Figure 2.

*Line 574. To obtain an estimation of spatial contributions to decoding performance, we performed a channel-searchlight analysis. For each EEG channel, we selected the nearest 4 neighbouring channels and performed the same decoding analysis as above, storing the accuracy at the centre channel, obtaining a time-varying spatial map of cross-validated decoding accuracies for each participant.*

**1.4** Figure 1B – the generalization maps are quite small - I would also push them to the same colorscale, requiring only one scale + freeing up space to enlarge them (and making them more comparable). It is unclear to me whether they were additionally masked by significance

We have changed the plots to make them clearer. The time range is now restricted to the main time window of interest (0-400ms) and they are plotted using the same colour map. They still have different scales because it is hard to see details of the orientation analysis otherwise, but now we include the scale for each plot. The generalisation plots are masked to show only time points with BF>10. This detail has been added to the figure caption. The full time generalisation maps are now shown in Supplementary Material.

**1.5**	C1/P100 – C1 and P100 are quite different, I found it surprising to be seen in the same sentence. I don’t think the authors can make strong claims about C1, their timing is comparably late, and C1 (origin likely in V1) is typically identified via cruciform model / peripheral location of stimulus

Thank you, we agree that C1 is not relevant here and we have taken it out.

**1.6**	L294: I did not understand the logic of this argument. The idea is to explain why in MEG we don’t see a second bump. But being able to measure frontal-alpha (or not, as apparently in MEG), should not matter in this case, given that the weights in Fig 1B are all strongly occipital. Maybe the authors could clarify this passage.

We were speculating that the second bump might be information that is carried via alpha frequency from frontal regions to occipital regions. It seems possible that this could be a plausible reason for reduced decoding even in occipital regions, but of course this is just speculation and worthy of future research. We have tempered this point in the Discussion.

*Line 331. Alpha oscillations have been implicated in feedback-related coherent visual processing (Chen et al., 2023) and notably, frontal alpha and theta oscillations are difficult to measure using MEG (Srinivasan et al., 2006). Frontal oscillatory activity might therefore play a role in high level visual representations after 150ms, in a potential feedback process that influences processing within occipital cortex.*

**1.7**	In L351 the paper argues that the orientation effect is so small, maybe because orientation is not an important feature. What I was missing was, that it could just be quite hard to measure in EEG due to cancellations within the fusiform gyrus. Thus, if you take the cruciform model, maybe activity over hemispheres (and top/bottom) cancels each other out, making it unmeasureable. It could then be, that showing a rotated stimulus only within one quadrant, that we can decode orientation quite fine – simply not if it is extended over the whole screen. Not sure what the authors can do with this, maybe there is some prior research on it?

It is possible that orientation effects are hard to measure due to activity cancellations, but it seems unlikely that this would disproportionately influence one feature and not the others. Anecdotally, we and others have found it quite difficult to decode orientation in the periphery. Furthermore, we see weak decoding from even the earliest stages of processing and it seems unlikely that activity cancellations within one region (e.g., fusiform gyrus) would be reflected throughout the whole time course. Overall, we still think our results point to coding of orientation being less robust than other features, potentially because local orientation is not crucial for form perception.

**1.8**	In relation to odd-one-out-triplet, I think this paper might be of interest <https://jov.arvojournals.org/article.aspx?articleid=2783900> (no expectation to include/cite that paper!)

Thank you for drawing attention to this interesting and very relevant paper. We have added a citation in the manuscript in the Discussion.

*Line 458. Previous work has used the odd-one-out triplet task to great effect in finding the dimensions underlying similarities in a large stimulus set (Hebart et al., 2020; Kunstle et al 2022).*

**1.9**	L451: Luminance levels (and environment) level might be interesting to some researchers

The room was dimly lit. Exact luminance values in the lab were not measured. We have added this detail to the Methods.

Line 536. “Participants viewed the stimuli on a 60 Hz ASUS VG236H 1920 x 1080 pixel gaming monitor from approximately 57cm away in a dimly lit room (exact luminance values were not recorded)”

**1.10**	L478: What monitor was used?

Monitor details been added to the methods.

*Line 536: Participants viewed the stimuli on a 60 Hz ASUS VG236H 1920 x 1080 pixel gaming monitor from approximately 57cm away in a dimly lit room (exact luminance values were not recorded)*

**1.11** In general, I could not see in the code or paper that the monitor delay was adjusted. This is quite important given the discussion of all the onset-timing-effects. Same is true for potential filtering delays.

This is an important point. Our lab setup is calibrated to have a negligible delay between image presentation and the trigger. In our PsychoPy script, the trigger is sent immediately after the image flip is sent. We did not assess the monitor delay specifically during this study, but we have for another studies in the same lab with similar presentation code and are quite certain that the timing is accurate (i.e., less than 10ms delay). With respect to filtering delays, any filter can somewhat distort the temporal estimates of different processes. We use the EEGLAB default filter which is a zero-phase non-causal filter, so should result in minimal time smearing (or at least we can expect that any distortions are fairly standard in the field). Overall, we acknowledge that estimates of timing can vary across laboratories and analysis pipelines, so our main points relate to relative differences in timing across stimulus conditions, rather than the absolute values. We have added some details to the Methods and a caveat in the Discussion.

*Line 541. An event trigger was sent over the parallel port at every stimulus onset event.*

*Line 551. Basic processing was performed: data were filtered using 0.1 Hz high pass and 100 Hz low pass filters, using EEGlab default Hamming windowed sinc FIR zero-phase filters.*

*Line 313. We acknowledge that absolute timing estimates can vary across studies due to specific laboratory and analysis details, so we focus on the differences in timing between conditions.*

**1.12**	L488: Targets occurred more often in one SOA condition to the other, is that correct? Maybe making this transparent, if true, is helpful to some.

Each sequence contained the same number of images and targets (on average), regardless of SOA condition. This meant the targets were closer together in time in the fast SOA 20hz sequences. We have clarified this in text.

*Line 545. Targets occurred 2-4 times per sequence.*

**1.13**	SOA was confusing to me, as asynchronisity implied to me a jitter. Stimulus-Onset-Interval might be clearer?

We have removed the term ‘SOA’ from the manuscript..

**1.14**	L494: Quite some details on the used filter is missing.

We have added this in the Methods.

*Line 552. using EEGlab default Hamming windowed sinc FIR zero-phase filters.*

**1.15**	There is no correction/control for eye-movements, even though in some experiments stimulus conditions can be decoded from fixational eye movements alone. I don’t particularly think this is a big issue here, given the feature importance showing little weight in frontal channels – but something worth discussing. E.g. Mostert showed this in MEG with orientation effects <https://www.ncbi.nlm.nih.gov/pmc/articles/PMC6179574/> .

We believe our design itself minimises eye movements because stimuli are presented rapidly, and at fixation. Unlike the Mostert study, there was no task on the stimulus, and no probe. Furthermore, fast eye movements take ~100ms to initiate (Fischer and Ramsperger, 1984), whereas the longest stimulus duration was 133.33ms in our design. Therefore, any eye movements are likely to be irrelevant to the early stimulus related information extracted from the EEG signal. We have added a note about this in the Methods.

*Line 555. We did not control for eye movements or other artefacts in the analyses because multivariate classification is robust to random noise and artefacts (Carlson et al., 2020). Furthermore, our design minimises eye movements because all images were presented at fixation, for short durations and in random order.*

**1.16**	The circular nature of orientation was not used in this study, potentially leading to lower than possible decoding abilities.

It is true that we did not specifically model the circular nature of orientation in our decoding analyses, but we also did not model the linear/ranked nature of the other features. The classifiers were 4-class multi-class decoders, which means they are trained to optimally separate the 4 classes and do not take into account the relationship between the classes.

The RSA models more explicitly incorporate the within-feature relationships (such as the circular nature of orientation) and still showed a decrement for orientation relative to the other features, so we are not convinced circular models would conclude anything different.

**1.17**	One Question that was raised during reading (outside of scope of review – but still interesting to me, please ignore if you don’t have time to engage!)

-	The n+1 / n+2 decoding ability, are these sequence effects? That is, if sf = 1 is followed by sf = 1, can we decode the latter one better or worse? I could envision it going both ways. In general, further I wonder if the “temporal code” changes (similar to evoked/sustained response of the cortex – see e.g. <https://doi.org/10.1016/j.celrep.2023.112614> )

This is an interesting question, but our design can not be used to answer this. We had no exact repeats of the same stimulus but there were repeats of feature levels (eg sf1ori1 followed by sf1ori2). We would predict decoding would be worse on exact stimulus repeats in line with repetition suppression but perhaps there would not be an effect for feature repeats when other features change.

**Reviewer #2:**

In this study, the authors apply neural decoding to evoked potentials (measured via EEG) while human participants (n = 16) viewed Gabor stimuli that systematically varied in contrast, orientation, spatial frequency, and color while engaged in a distractor task at fixation. While the authors explore the decodability of those Gabor features by themselves (replicating previous work in the temporal domain), their main focus was on the dynamics of the decoding results based on the interactions and conjunctions of those features. They also relate their decoding results to the behavioral performance of an independent group of participants engaged in a simple ‘odd-one-out’ task using the same stimuli. The results confirmed previous work (differences in EEG and MEG signal sources aside) and revealed interesting decoding results as a function of different feature conjunctions that may support earlier results from behavioral psychophysics. The also show that the backward masking inherent in their EEG stimulus paradigm seemed to have little impact on feature decodability. Overall, I found the manuscript to be clearly written and relatively straightforward to follow. I do have some questions/concerns for the authors to consider (given below in no particular order).

**2.1**	The question of how ‘low-level’ feature encoding contributes to task performance (or perceptual experience) in humans is not new. And, despite the authors’ claim, a significant amount has been learned about this question from paradigms ranging from EEG, MEG, to fMRI. The authors paint a picture that leads the reader to believe that we know very little in the way of an answer to that question – there are many in the visual neuroscience community that would strongly disagree (at least in terms of how the study is motivated in the introduction). The authors would be well advised to recast their motivation for the current study in light of the recent (and semi-recent) EEG work from the labs of Michelle Greene, Iris Groen, Bruce Hansen, Assaf Harel, and Steven Scholte. All have contributed insight into the temporal dynamics of filter based features (such as those explored in the current study) in support of scene perception and related task performance -- all with stimuli and tasks that are much more ecologically valid than what is employed here. Related to that, the authors note that human neuroimaging has mainly focused on low-level perceptual psychophysics, which is incorrect (see recent work by those, and others, noted above). In fact, one could easily classify the psychophysical paradigm employed in the current study as a ‘low-level’ psychophysical task.

Thank you, we take this point and agree that the Introduction could benefit from better placement of this study in the current state of the field. We have added more citations and more clearly stated how our study extends knowledge in this area.

*Line 39. However, there are still many open questions regarding how visual feature coding relates to perception. More recent work has investigated the link between low-level neural responses and perception, albeit in different contexts and not separating individual basic features. In one study, stimulus similarity judgements were found to be tightly related to early visual brain responses for geometric patterns (Wardle et al., 2016). Other work showed that low-level stimulus features had good concordance with neural responses, but were not predictive of perceptual judgements of scenes (Greene and Hansen, 2020). However, early visual responses and low-level models in these studies comprise multiple visual features that could have different contributions to perception. To really characterise the transformation of visual information across the hierarchy, we need to answer how these basic building blocks of vision (e.g., orientation, spatial frequency, contrast, colour) are combined and subsequently contribute to the overall percept of a stimulus.*

*Line 80. In addition to understanding how individual visual features (and combinations of these features) are encoded in the brain, a critical aspect that has remained a challenge is understanding how encoding of individual features informs our perception of the stimuli. A recent development in the field has been to explore more deeply the extent to which neural coding informs conscious perception. High level information has been shown to be predictive of behaviour for object categorisation (e.g., Carlson et al., 2014; Grootswagers et al., 2018; Mur et al., 2013; Ritchie et al., 2015) and scene perception (e.g., Greene and Hansen, 2020; Groen et al., 2018; Harel et al., 2020; Seijdel et al., 2020). It seems quite clear that neural responses to complex information such as form and category drive behaviour, but is the same true for the first stage of processing in our visual system? For example, are behaviourally relevant features coded more strongly in the brain? How fast do we get behaviourally-relevant coding? One possibility is that even the earliest coding of features is relevant for behaviour, but alternatively perhaps perception is more reliant on later processes.*

**2.2** Mapping the temporal dynamics of simple visual features when simultaneously presented (e.g., a color Gabor of a certain orientation, spatial frequency, and contrast) is an important undertaking. However, I can’t help but wonder how much of any of what is reported here would hold if the stimuli were more ecologically valid. Our visual world is filled with structures that simultaneously activate a broad range of visual neurons that vary in their feature tuning. Decades of studies have shown that those neurons interact – resulting in myriad nonlinear competitions for which population will contribute to the percept and/or support a given task. This is why, at approximately the turn of the century, many in the field shifted away from simple Gabor stimuli in favor of more complex stimuli – then seeking models composed of various filters to decompose those stimuli in a way that maps onto neural or behavioral response variance. The linkage to behavior in the current study is nice, but again, the task itself is just a discrimination task between Gabors. All that said, I’m not saying that the current study has nothing to offer, just that great caution should be taken (explicitly stated) in terms of just how much of what is reported here will hold when it comes to how the system actually works in the real-world.

We agree with the point that there is a lot to be learned from studying complex stimuli, particularly for understanding ecologically valid behaviour - most of our recent work in fact has been using real world object stimuli. However, in this study we took the tack of using relatively simple stimuli and having complete control over their features, which has the advantage of explicitly being able to link basic visual features with perception. Though they are not ecologically valid, there is still a lot we can learn from Gabor stimuli given that the early visual system is explicitly tuned to these types of stimuli. We have added a point on this in the Discussion.

*Line 476. Here we used stimuli that are highly controlled and have little semantic meaning, which allowed us to carefully study the dynamics of individual feature representations as well as nonlinear interactions between features and their relationship to overall form perception. Our approach varies from the recent push to study complex stimuli, which has yielded much insight into ecologically valid perception (Allen et al., 2022; Harel et al., 2020; Hebart et al., 2019; Mehrer et al., 2021), yet our ability to control the stimulus features conveys an advantage not yet possible (or much more difficult) with real world stimuli.*

*Line 492. One open question that remains is whether the neural-behaviour relationship varies according to how much the stimuli are reliant on semantic processes.*

**2.3** Related to the above, the 1980s (and late 1970s for that matter) are filled with psychophysical studies that used Gabors (or sinusoidal gratings) composed of systematically varying features – all attempting to elucidate how such features interact (the primary focus of the current study)….far too many to list here, but it would at least be nice to know how much of what is reported here supports some of that earlier work.

There are indeed many studies that used Gabors to study feature processing. In the revision, we have added crucial references and reviews in the Discussion to better place our observations in the existing literature.

*Line 406. “These results fit well with the larger history of perceptual interactions documented in the psychophysics literature focused on contrast sensitivity functions, where spatial frequency sensitivity varies across contrast (Campbell and Robson, 1968; De Valois, 1978; Mullen, 1985). Recent work has found evidence that red/green gratings have higher contrast sensitivity than yellow/violet, which is supported by our findings that spatial frequency and contrast were more highly decodable for the bluer/redder gratings than the green/purple gratings (Taveras-Cruz et al., 2022; Wuerger et al., 2020). Interactions for the other features have been documented but have not been studied extensively (De Valois and De Valois, 1980; Taveras-Cruz et al., 2022).”*

**2.4** Please provide more detail concerning the group-level analyses – exactly how were the data from each participant entered into the analysis (give an appendix if it’s already made clear in an earlier publication by this group). It doesn’t seem like the decoding was run on an individual-by-individual participant basis – the confidence intervals are strikingly narrow, suggesting that it wasn’t). If not, why? It would be very informative to the audience to get a good sense of the extent to which the reported results are observable at the individual level. Similarly, it would be good to report signal quality tests on each participant (e.g., split-half prediction or something of that ilk). The differences in cortical folding can lead to certain sources being obscured on the scalp and add unnecessary noise to the analyses. No mention of signal quality checking is given here.

We agree there were some missing details and have expanded upon the analysis details in the Methods. Briefly, decoding was run on an individual participant basis, and then the mean was taken across the group. Consistency in decoding across participants was very high, which led to very small error bars on the plots. We now clarify that the error bars are standard error of the mean in the caption of each plot. We have also added an additional Figure that shows feature decoding per participant which shows the consistency across individuals (also in response to Reviewer 3’s comments).

*Figure R1/Figure 3. Feature decoding per participant for 6.67Hz condition. Information about all four features can be distinguished in highly similar time windows at the individual participant level, suggesting the observed feature coding results are highly reliable.*

**2.5** The EEG task presents Gabors to the fovea (extending only a degree or so into the periphery), so why such a low range of spatial frequencies (2.17cpd is the highest spatial frequency used here). Similarly, why did the authors choose to leave out the cardinal orientations? Might the odd reduction in decoding accuracy in color with decreasing spatial frequency be accounted for by this somehow? The color system is largely lowpass, so I found that result more confusing than interesting.

Ideally, we would have tested more levels per feature and different ranges. However, the 4 levels for 4 features led to 256 unique stimuli, which is already pushing the limit of the number of stimuli we could test in a single EEG session. For spatial frequencies, we chose values that seemed separable by eye and did not exceed the peak of the contrast sensitivity function for ease of interpreting interactions with other features. Regarding the choice of orientations, we left out the cardinal orientations to avoid effects being driven by the cardinal bias. Moreover, previous work has characterised decodability of cardinal versus oblique orientations (Pantazis et al., 2018).

Finally, the colour x spatial frequency interaction does seem unexpected at face value when considering studies into contrast sensitivity of chromatic gratings show lowpass responses (e.g., Mullen, 1985). However, our finding that colour discrimination increases with increasing spatial frequencies does fit with work showing that colour sensitivity of V1 neurons is spatially bandpass (Johnson, Hawken & Shapley, 2001). Our results might indicate a previously unexplored effect at lower spatial frequencies. We have added a sentence into the Discussion about the limitations of our chosen feature values.

*Line 485. It should be noted that we only had four stimulus levels of each feature (e.g., four different spatial frequencies), and the results might be somewhat dependent on these chosen values, so future work could interrogate this feature space further.*

**2.6** Why are there two different SOA paradigms used in the current study? Some justification (or at least some rationale) should be given in the manuscript.

Different presentation rates in RSVP result in different depths of processing. For example, slower versus faster presentation rates result in better behavioural detection of images (e.g., Potter et al 2014) and stronger neural representations (Robinson et al 2019). Here, we expected that feature coding, as an early visual process, would be resistant to presentation rate, so we included the two rates to test this. We show that feature coding is not dependent on the speed of presentation, which shows the robustness of feature coding. We have added more motivation to the Introduction.

*Line 96. To assess to what extent feature processing, as an early visual process, is dependent on processing time, we used different presentation rates to target different depths of processing (McKeeff et al., 2007; Robinson et al., 2019). Previous work has shown slower presentation rates result in higher behavioural accuracies (e.g., Potter et al 2014) and stronger neural representations (Robinson et al 2019) than faster rates. Here, stimuli were presented in fast sequences at 6.67 Hz or 20 Hz, which bias to higher and lower stages of processing, respectively (Collins et al., 2018; Grootswagers et al., 2019a, 2019b; Robinson et al., 2019).*

**2.6a** Also, how large was the bullseye for the distractor task in the EEG experiment?

The fixation bullseye was 0.6 x 0.6 degrees of visual angle. This has been added to the Methods.

*Line 544: “The task was to detect when the fixation bullseye (presented centrally at 0.6 degrees visual angle) changed to a filled circle target and respond using a button press.”*

**2.7** Crucial details about the monitor have been left out (outside of stating that it has a temporal resolution of 60Hz). Please provide the specs on the monitor. Also, I assume that the stimuli were linearized – please mention that. If it wasn’t, then the results will need to be considered in the context of the stimuli not being linear with respect to luminance.

We added the monitor details in the revised manuscript (see point 1.10). Stimuli were not perfectly linearised in terms of luminance. Our methods do not depend on linearised stimuli; RSA for example focuses on the rank-order of the feature levels and compares across neural responses and behaviour using Spearman correlations. Thus our results or conclusions are unlikely to change for linearised stimuli.

**2.8** For the EEG data, why were they down sampled to 250Hz? Also, given the lowpass filter had a cut off at 100Hz, I assume that there was no electrical noise? If a notch filter was used, it should be reported.

EEG data were downsampled to 250Hz to reduce computation time and in line with previous work on time course decoding (Grootswagers et al., 2017). We did not remove electrical noise or do any artefact correction because these steps make little difference to classification and allow us to maintain a very basic, reproducible pipeline. We have added more detail to the Methods.

*Line 551. “Basic processing was performed: data were filtered using 0.1 Hz high pass and 100 Hz low pass filters, using EEGlab default Hamming windowed sinc FIR zero-phase filters. Epochs were constructed from 100ms prior to 600ms after each image presentation and downsampled to 250 Hz. No electrode interpolation or artefact rejection was performed (cf. Delorme, 2023).”*

**2.9** Given that participants in the EEG experiment and those in the psychophysical experiment were doing different tasks, I don’t think it’s appropriate to make the connection between poor classification for orientation and poor predictability of perceptual similarity. The behavioral participants clearly prioritized some features of others in the triplet tsk – the same cannot be said for those in the EEG experiment doing a task that didn’t require them to use the Gabor features at all.

We understand that it may seem counterintuitive, but it is a common approach to compare neural representations in one group to behaviour in another, even using different tasks. We find reliable correlations between featural information obtained from neural data measured in the EEG session and perceptual similarity results obtained in different participants. It is notable that the two groups performed different tasks, but this effect is in line with many previous studies that show notable correspondence between neural responses and behaviour (e.g., Bankson et al., 2018; Cichy et al., 2019; Contini et al., 2020; Grootswagers et al., 2022; Mur et al., 2013; Wardle et al., 2016), suggesting that the neural coding of visual information is inherently formatted for behavioural read-out regardless of whether the behaviour is being performed. We agree that the differences in tasks put a limit on what we can conclude about the trends in results for each study, but we do not think it is inappropriate to speculate that poorer coding (of orientation relative to other features) might correspond with differential prioritisation of features in the triplet task (see also our changes in response to point 3.4).

*Line 471. Previous work that made similar comparisons between neural data and behavioural data from separate participants has shown that perceptual similarity correlates with neural responses (e.g., Bankson et al., 2018; Cichy et al., 2019), even to the point that behaviour can explain the most variance in neural responses (e.g., Bankson et al., 2018; Contini et al., 2020; Grootswagers et al., 2022; Mur et al., 2013; Wardle et al., 2016), indicating that perceptual decision-making might be based on a direct behavioural read out of the patterns of stimulus-based neural responses*

**Reviewer #3:**

In this manuscript, the authors use an EEG paradigm to trace the neural dynamics of visual features and their interactions along visual hierarchy, using the Gabor stimuli displayed in a rapid sequence (6.67Hz vs. 20Hz) that each stimulus parametrically varies in feature combinations along 4 dimensions (orientation, spatial frequency, color, and contrast). Based on a multivariate decoding analysis, they then study how single feature and their non-linear conjunction are dynamic represented in visual cortex. Additionally, they try to explore the behavioral relevance of these neural signatures using representational similarity analysis (RSA), in which the behavioral matrix (perceptual similarity between the tested Gabors) is calculated using a separately data set tested on a new group of participants.

Overall, the general approach taken in this paper – applying different classifiers to the same set of parameterized stimuli – is promising to study the neural dynamics of visual information processing in the brain. The question the authors set out to investigate is relevant and the manuscript is well written. However, I have multiple comments listed below. To preview, my main concern is about the limited knowledge that this paper can add to the field. There is little novel insight that change or add to our understanding of the Information processing dynamics and computations along the visual hierarchy.

Major comments:
**3.1** The authors trace the coding dynamics of each feature (orientation, SF, colour, and contrast) in the brain and show a temporal profile displaying two representational peaks, one around 100ms and the other 170m. Such typical effects have been observed in so many EEG/MEG studies involving visual recognition, categorization or with just passive viewing paradigm, and at both object/image level and at decomposed single feature level. Additionally, it has been well demonstrated that multiple features of a given stimulus are represented along the visual hierarchy in parallel, with the early peak (~100ms) activates the early visual cortex (same as the reported results in this manuscript, see Figure 2 topological map) and the later peak (~170) propagates towards the occipital-temporal junction (the authors did not check this in their manuscript). Though the authors state that their analysis shows different coding dynamics of each feature in terms of onset/peak time and temporal generalization pattern based on the group average analysis (in Figure 2), I quite doubt the reliability of such results due to the very high individual difference in single feature representations in the brain found in previous and my own MEG studies. This issue can be probably resolved by adding individual-level analysis together with prevalence analysis, even so this paper only shows a phenomenon rather than provides explanation of underlying mechanism. And we still don’t know why our brain represents these features differently, what is their perceptual or cognitive significance? Taken together, the feature-level dynamics in early visual cortex revealed in current study can be well predicted as which has been well-investigated in previous studies.

We thank the Reviewer for these points. We agree that the temporal profile of our results fit very well with previous work, including our own. We have emphasised the novel aspects of our study in the revised manuscript (see also points 2.1 and 2.3).

The searchlight results are now shown in Supplementary Material. We find the spatial distribution of both the first and second peaks are focused on central occipital regions. This suggests the second peak might be recurrent processing or feedback rather than a propagation in information to temporal regions, though with the spatial resolution of EEG it is difficult to be sure.

Reviewer 2 also raised the point about individual variability (see point 2.4). We actually find extremely reliable patterns of results for each participant and have included these plots in the manuscript (see new Figure 3). These results speak to the power of our approach, and show quite convincingly that there are different dynamics for different features.

*Figure R1/Figure 3. Feature decoding per participant for 6.67Hz condition. Information about all four features can be distinguished in highly similar time windows at the individual participant level, suggesting the observed feature coding results are highly reliable.*

3.2. The authors find interesting asymmetric interaction patterns between the coding of different features. However, I have problem of understanding these results (Figure 3). How such representational interactions emerge during the passive viewing task? Why our brain displays such asymmetric feature interactions? To answer the first question, the authors should locate where and when such interaction happens in the brain at least at channel level (or better at source level using source reconstruction analysis), which may provide some evidence to promote our understanding. However, for the later question in above, I don’t think the authors can provide good answer based on their current design. What I wonder is whether the observed results constitute a general property of perceptual processing (e.g., generalize to other stimuli type that can be decomposed into similar feature dimensions, and to tasks with different cognition demands such as passive viewing, recognition, categorization) or are very task-specific (e.g., constrained by the paradigm used in current study). Even it was the later, we still need to have a mechanism explanation over the observed phenomenon (as I raised in comment 1). However, based on their current experimental manipulations (the 6.67Hz vs. 20Hz stimuli display rate), I don’t think it targets to understand the nature of the feature interactions. So again, the purely interaction phenomenon revealed in current results add very limited knowledge to the related research fields.

We interpret our findings to reflect robust neural responses that are task-independent. We and others have found that robust visual representations emerge during passive viewing (for a review, see Robinson et al 2023), so it seems plausible that basic feature interactions would also emerge without a task. We cannot speak to task effects because we did not vary task in the EEG design. However, we do show that the interactions are largely invariant to presentation rate, with the same trend in results for the 6.67Hz and 20Hz sequences. Furthermore, we show that perceptual decisions in an odd-one-out task correlate with these passive viewing representations, suggesting that these processes we are measuring with EEG are fundamental for subsequent behaviours, even if the task is different. In terms of the asymmetric feature interactions, we find these very interesting but of course they require further study to interrogate further. Nevertheless, they appear to be reliable because we see the same interactions at the two different frequencies.

The manuscript is largely concerned with the temporal dynamics of feature and conjunction processing, thus we show the time courses and tabulate the onsets and peaks of the emergence of these representations. In terms of the spatial extent of these representations, we show the searchlight results for the individual features but have not gone any further because we think that EEG is not the best method to investigate where these processes are happening in the brain.

While we do not propose a new mechanistic explanation for feature and conjunction coding in the brain, we would still argue that our investigation into feature processing provides a great deal of insight into the temporal dynamics of neural population coding in the human brain. We provide an in depth look at feature interactions both in the brain and behaviour, extending previous work on colour and form processing. Finally, we draw links between processing of specific basic visual features and perception, so we believe the work is novel and important.

In the revision, we have rewritten sections in the introduction and discussion to reflect these points.

3.3 The authors designed a very clever classifier to investigate the feature conjunctions in the brain. Based on my understanding, the classifier is an XOR solution so that it requires a (non-linear) integration of 2 features together to generate an output. I quite appreciate this idea. However, tracing low- (and high-) level feature integration dynamics is not new in human EEG/MEG studies (see K Jaworska et al., 2022, elife, with a similar pattern but with much better spatial-temporal resolutions) which limits my enthusiasm for this result. Also, as the analysis approach mixes many different components, I found it is very difficult to interpretate and evaluate their conjunction results. For example, for each feature-pair conjunction, it averages across 36 combination possibilities; however, due to a different feature-by-feature interaction pattern, I would expect different temporal profile (e.g., onset/peak/amplitude) of each combination possibility, and then averaging them out can lead to some biased conclusion. Additionally, the sustained conjunction decoding curves and their multiple peaks (see Figure 4B) suggest different brain sources (e.g., brain regions/voxels) contributing to the feature integration at different processing stages; however, the authors mix everything up and then the results become very uninformative to me. Taken together, I doubt the reliability of this conjunction results. For example, the authors show a counter-intuitive result to me, i.e., early feature conjunction before the individual feature representation (see Figure 4B, colour*contract and SF*contrast panel).

Thank you, this is a useful comment. To assess the reliability of the conjunction analyses, we looked at each of the 36 combination possibilities for each feature pair conjunction (Figures R2 and R3, added to supplementary materials). The peak decoding magnitude differed across combinations, but the trend in results appeared quite consistent in terms of the dynamics. For example, for the reliable feature conjunctions (colour x contrast, SF x contrast, SF x colour, orientation x SF), the different curves peaked at similar times. Thus, we consider the conjunction decoding results shown in the manuscript (the mean of these 36 curves) to be an accurate representation of feature conjunction coding for each pair of features.

Regarding the brain sources underlying the integration, we focus on the temporal dynamics rather than the spatial location of coding. Our whole brain decoding approach does indeed mean that different sources can be contributing to different time periods. Interestingly, the new searchlight plots for individual feature coding shows most feature information is clustered around central occipital (*early*) visual regions, even at later time points. However, properly assessing the spatial dynamics was beyond the scope of the current study.

*Line 335: “the searchlight analyses showed the most feature information was constrained to central occipital electrodes for both the first and second peak of decoding (see Supplementary Material)”*

To clarify the result in Figure 4B, there is a difference in the size of the confidence intervals for the onset and peak estimates of conjunction coding which might be causing the confusion. Importantly, for colour x contrast and SF x contrast, the conjunction *onsets* were later than the onsets of individual feature representations, as expected. The peak estimates, however, were a bit noisier, likely due to multiple similarly sized peaks, meaning the confidence interval of peak coding included the peak of the individual features. Importantly, the peak estimates were still later than the onsets of the individual features. The same is true for all reliable conjunctions (i.e., every combination except orientation x contrast and orientation x colour, which have limited evidence for conjunction coding). Therefore, from our results we can conclude that conjunction coding, when present, occurred *after* individual feature coding.

***Figure R2.*** *Mean feature conjunction decoding across participants for 6.67Hz condition, shown for each combination of feature levels. Plots show decoding of each of the 36 specific feature combinations. For example, in the colour x contrast plot, there is one line for decoding [colour1/contrast1 and colour 4/contrast4] vs [colour1/contrast4 and colour4/contrast1], and another line for [colour2/contrast1 and colour3/contrast2] vs [colour3/contrast1 and colour2/contrast2]. There is variability in the magnitude of decoding for each contrast, but the general dynamics look very similar within each plot across the 36 comparisons, indicating the conjunction analysis is reliable.*

***Figure R3.*** *Mean feature conjunction decoding across participants for 20Hz condition, shown for each combination of feature levels. Plots show decoding of each of the 36 specific feature combinations. Again, the dynamics of conjunction coding look similar for each contrast.*

3.4. The current design does not have experimental power to investigation the behavioural relevance of feature coding along visual hierarchy, for 4 reasons: 1) they across-corelate the data obtained in 2 independent experiments, that 2) recruit different participants to 3) perform very different tasks (one is passive viewing with an orthogonal stimuli-irrelevant task, and the other is a stimulus-based similarity judgement that requires strong feature processing demand) in 4) different experimental environments (online vs. in scanner) Due to the high individual variability in their ability of Garbor-related feature processing and the significant manipulation of task demand on both the early low-level feature processing in occipital regions and late high-level feature integration in ventral temporal regions (together with frontal modulation), I don’t think the reported data cannot support the behavioural-relevance claims made in this paper.

We understand that it may seem counterintuitive, but it is a common approach to compare neural representations in one group to behaviour in another. Many previous studies have shown that stimulus-related behaviour in one group of participants is highly correlated with neural stimulus representations in another group (e.g., Bankson et al., 2018; Cichy et al., 2019; Contini et al., 2020; Grootswagers et al., 2022; Mur et al., 2013; Wardle et al., 2016). The theory behind such findings is that visual stimulus representations are so robust and drive perceptual behaviour in such a way that group stimulus-based effects are stronger than inter-individual differences. Indeed, for neural representations, the estimate of the best model is the group average, which typically has the highest correlation of all models, and is often called the “noise ceiling”. We consider the method of comparing neural representations and behaviour across groups to be a strength, because it shows that perceptual representations are not specific to individuals or tasks or environments, but a robust coding strategy of the brain. We have added additional information about this approach to the Discussion.

*Line 471. Previous work that made similar comparisons between neural data and behavioural data from separate participants has shown that perceptual similarity correlates with neural responses (e.g., Bankson et al., 2018; Cichy et al., 2019), even to the point that behaviour can explain the most variance in neural responses (e.g., Bankson et al., 2018; Contini et al., 2020; Grootswagers et al., 2022; Mur et al., 2013; Wardle et al., 2016), indicating that perceptual decision-making might be based on a direct behavioural read out of the patterns of stimulus-based neural responses.*

3.5. An important manipulation in this study is using RSVP paradigm to control the displaying rate of the visual stimuli in 2 conditions (6.67Hz vs. 20Hz), however the authors provide no explanation why they introduce such manipulation. In their introduction, they should include scientific motivation of such design with appropriate cited references, to explain why they choose these two conditions not others, and what kind of hypothesis they can test based on such Hz (i.e., different ISI-SOA combination) settings. Without this information, it’s very difficult to interpretate and evaluate their null effects reported in current study.

Thank you, we agree the presentation rate manipulation needs more explanation. Different presentation rates in RSVP result in different depths of processing. For example, slower versus faster presentation rates result in better behavioural detection of images (e.g., Potter et al 2014) and stronger neural representations (Robinson et al 2019). Here, we expected that feature coding, as an early visual process, would be resistant to presentation rate, so we included the two rates to test this. We show that feature coding is not dependent on the speed of presentation, which shows the robustness of feature coding. We have added more motivation to the Introduction.

*Line 96. To assess to what extent feature processing, as an early visual process, is dependent on processing time, we used different presentation rates to target different depths of processing (McKeeff et al., 2007; Robinson et al., 2019). Previous work has shown slower presentation rates result in higher behavioural accuracies (e.g., Potter et al 2014) and stronger neural representations (Robinson et al 2019) than faster rates. Here, stimuli were presented in fast sequences at 6.67 Hz or 20 Hz, which bias to higher and lower stages of processing, respectively (Collins et al., 2018; Grootswagers et al., 2019a, 2019b; Robinson et al., 2019).*

Minor comments:
3.6 There are 4 levels of each feature dimension. The authors should explain how they choose the feature range with 4 equally sampled level, e.g., based on parameters used in previous studies or based on a psychophysical piloting? I notice they did not perceptually calibrate the feature levels per participant, which exacerbates my concern about the potentially large individual differences (related to my major concerns raised in point 1 and 4).

We tried to pick a representative range of feature levels keeping in mind that we had a limited (though large) number of stimuli. We chose 4 levels to drive differences and assess coding across a dimension, but the levels were not exhaustive. As can be seen in the new Figure 3, individual feature decoding is highly reliable and we have added a point in the Discussion that future work could interrogate this space further.

*Line 485. It should be noted that we only had four stimulus levels of each feature (e.g., four different spatial frequencies), and the results might be somewhat dependent on these chosen values, so future work could interrogate this feature space further.*

3.7. The authors use “perceptual experience” in their abstract and throughout the manuscript. It’s not clear to me what the *perceptual experience* means in their work. I believe researchers in the field of object categorization vs. perceptual learning will give very different explanation for this term.

In the revision, we have replaced this term to avoid this confusion.
